# Neurologic involvement in seronegative primary Sjögren’s syndrome with positive minor salivary gland biopsy: a single-center experience

**DOI:** 10.3389/fneur.2023.1174116

**Published:** 2023-06-09

**Authors:** Yoji Hoshina, Ka-Ho Wong, Jonathan Galli, Rae Bacharach, Julia Klein, Dorota Lebiedz-Odrobina, John W. Rose, Bryan Trump, Christopher Hull, John E. Greenlee, Stacey L. Clardy

**Affiliations:** ^1^Department of Neurology, University of Utah, Salt Lake City, UT, United States; ^2^George E. Wahlen Veterans Affairs Medical Center, Salt Lake City, UT, United States; ^3^Department of Neurology, Penn State Health, Hershey, PA, United States; ^4^Department of Medicine, Division of Rheumatology, University of Utah, Salt Lake City, UT, United States; ^5^School of Dentistry, University of Utah, Salt Lake City, UT, United States; ^6^Department of Dermatology, University of Utah, Salt Lake City, UT, United States

**Keywords:** seronegative, primary Sjögren syndrome (pSS), nonspecific neurological symptoms, lip biopsy, neuropathic pain

## Abstract

**Objective:**

To assess the demographics, neurologic manifestations, comorbidities, and treatment of patients with seronegative primary Sjögren’s syndrome (pSS).

**Patients and methods:**

We conducted a retrospective chart review on patients with seronegative pSS evaluated by a neurologist at the University of Utah Health between January 2010 and October 2018. The diagnosis was based on characteristic symptoms, positive minor salivary gland biopsy according to the American-European Consensus Group 2002 criteria, and seronegative antibody status.

**Results:**

Of 45 patients who met the study criteria, 42 (93.3%) were Caucasian, and 38 (84.4%) were female. The patients’ mean age at diagnosis was 47.8 ± 12.6 (range 13–71) years. Paresthesia, numbness and dizziness, and headache were noted in 40 (88.9%), 39 (86.7%), and 36 patients (80.0%), respectively. Thirty-four patients underwent brain magnetic resonance imaging. Of these, 18 (52.9%) showed scattered nonspecific periventricular and subcortical cerebral white matter T2/fluid-attenuated inversion recovery hyperintense foci. Twenty-nine patients (64.4%) presented to the neurology clinic prior to pSS diagnosis, and the median delay in diagnosis from the first neurology clinic visit was 5 (interquartile ranges 2.0–20.5) months. Migraine and depression were the most common comorbidities in 31 patients (68.9%). Thirty-six patients received at least one immunotherapy, and 39 were on at least one medication for neuropathic pain.

**Conclusion:**

Patients often display various nonspecific neurological symptoms. Clinicians should express a high degree of skepticism regarding seronegative pSS and consider minor salivary gland biopsy to avoid delaying diagnosis, as undertreatment can affect patients’ quality of life.

## 1. Introduction

Primary Sjögren’s syndrome (pSS) is a systemic autoimmune disorder, with a female predominance (9:1) and a peak incidence at approximately 50 years of age (1). It is commonly characterized by xerophthalmia and xerostomia, although other systemic and organ-specific manifestations may occur (1).

Neurologic involvement has been reported to range from 8.5 to 70% of pSS cases; this wide range is owing to the diverse diagnostic criteria of pSS, along with different definitions of neurologic symptoms and dissimilar availability of neurophysiological diagnostic testing resources (2–4). Although the prevalence of brain magnetic resonance imaging (MRI) abnormalities in patients with pSS remains unclear, MRI abnormalities have been reported with and without clinical evidence of central nervous system involvement (5). These include abnormalities typically visible on T2-weighted imaging (T2WI) or fluid-attenuated inversion recovery (FLAIR) sequences, raising concerns of possible multiple sclerosis (MS) (6). While neurologic involvement in pSS has been studied, it is still likely under-recognized, especially when initial symptoms lead to neurologic consultation prior to rheumatologic consultation or diagnosis. For example, neuropathic pain contributes to a delay in diagnosis, especially when sicca symptoms are mild (7), and neurologic manifestations may precede other pathognomonic Sjögren findings in 25–60% of cases with a mean delay of 24 months to pSS diagnosis (3).

Detection of anti-Ro/SSA and anti-La/SSB antibodies has long been used as the major diagnostic test for pSS and is included in the American-European Consensus Group 2002 criteria (8). However, these tests may be negative in 25–33% of patients (1, 9). pSS can also be diagnosed in the absence of anti-Ro/SSA or anti-La/SSB antibodies with positive histopathological evidence via minor salivary gland biopsy. In such cases, patients are diagnosed with seronegative pSS (7, 10, 11). Differences in clinical features between seropositive and seronegative populations are still controversial. For example, the Sjögren’s Big Data Project of 10,500 patients reports that patients diagnosed with anti-Ro/SSA or anti-La/SSB antibodies have lower mean age at the time of diagnosis and a higher frequency of constitutional, renal, cutaneous, or hematological manifestations, which suggest that seronegative pSS may be a milder form of the disease (11). However, a more recent study comparing seronegative and seropositive patients reported similar demographic features between both groups (including age at diagnosis and sex distribution) and laboratory findings (except for antibodies) (7). None of these studies adequately characterize the neurologic manifestations observed in patients with seronegative pSS. Hence, we conducted this single-center study to assess the demographic and neurologic symptom profiles in this patient cohort.

## 2. Patients and methods

We performed an electronic database search of the University of Utah Health records between January 1, 2010, and October 31, 2018, to identify all patients with a positive minor salivary gland biopsy and seronegative antibody status, who were ultimately diagnosed with seronegative pSS, and were evaluated at least once by a neurologist. First, we extracted data for all patients diagnosed with SS using the International Classification of Diseases Clinical Modification, 9th and 10th revision codes 710.2 and M35.00. Thereafter, we reviewed their serological tests and clinic visit records. Patients who met all the following criteria were ultimately included in our analysis: (1) American-European Consensus Group criteria (8) for the diagnosis of pSS, (2) absence of anti-Ro/SSA and anti-La/SSB antibodies with a positive minor salivary gland biopsy result, and (3) at least one neurology clinic visit for any neurological complaint. All patients with SS associated with other established autoimmune diseases, such as that related to systemic lupus erythematosus or rheumatoid arthritis (RA), were excluded from the analysis. Positive minor salivary gland biopsy was confirmed based on the Chisholm and Mason classification (grades 1–4), and only grades 3 and 4 were included (12). Data collected for each patient included demographic information, clinical features focusing on neurologic symptoms, reason for neurology clinic visits, comorbidities, laboratory data, and radiologic data. Treatment and outcome data were also collected. The treatment outcome was assessed based on documentation during the follow-up appointment. The autonomic nervous system was assessed through a series of tests, including the quantitative sudomotor axon reflex test, heart rate response to the Valsalva maneuver and deep breathing, and blood pressure and heart rate response to the head-up tilt test. Cognitive impairment was defined as a Montreal Cognitive Assessment (MoCA) score ≤ 25.

A total of 286 patients were diagnosed with SS, of whom 94 tested negative for anti-Ro/SSA and anti-La/SSB antibodies. Of the seronegative patients, 56 patients presented to the neurology clinic with at least one neurologic symptom. Nine patients were excluded owing to a clinical diagnosis of pSS without minor salivary gland biopsy, and two patients were excluded owing to at least one other established rheumatologic condition (one patient had RA and the other patient had both RA and limited systemic sclerosis).

### 2.1. Statistical analysis

Values of quantitative variables are expressed as mean ± standard deviation or median and interquartile ranges (IQR), and values of qualitative variables are expressed as percentages. All statistical analyzes were performed using R Statistical Software (version 4.1.2; R Foundation for Statistical Computing, Vienna, Austria).

### 2.2. Standard protocol approvals, registrations, and patient consent

The study procedures were approved by the local institutional review board of the University of Utah (IRB_00108537).

## 3. Results

Forty-five patients were ultimately included in the analysis for the study. Table 1 summarizes the demographics of the patient population. The mean age was 47.8 ± 12.6 (range 13–71) years. Thirty-eight patients (84.4%) were female, and 42 patients (93.3%) were Caucasian. Twelve patients (26.7%) had at least one first-degree relative with rheumatologic disease, including pSS in two cases (4.4%). All patients in this study had at least one of the sicca symptoms (40 patients had xerophthalmia, and 40 patients had xerostomia).

**Table 1:** Patient characteristics.

Characteristic	*n*	%
Sex		
Male	7	15.6
Female	38	84.4
Mean age at diagnosis of primary Sjögren’s syndrome, years (range)	47.8 ± 12.6 (13–71)	
Median follow-up, months (interquartile ranges)	19 (3–46)	
Ethnicity		
Caucasian	42	93.3
African descent	2	4.4
Asian	1	2.2
Family history in first-degree relative		
Autoimmune conditions	12	26.7
Primary Sjögren’s syndrome	2	4.4

Neurologic manifestations are summarized in Table 2. The most common neurologic symptom was paresthesia (*n* = 40, 88.9%), followed by numbness and dizziness (n = 39, 86.7%), and headache (*n* = 36, 80.0%). Among the patients who had paresthesia, eight were diagnosed with small fiber neuropathy (six patients showed positive skin biopsy and two patients were diagnosed based on clinical symptoms, evidence of small fiber neuropathy on an electrophysiologic test, and normal nerve conduction test.) Among the 34 patients who underwent brain MRI during this period, 18 (52.9%) showed scattered T2WI/FLAIR hyperintensities in the periventricular and subcortical white matter, two of whom were eventually also diagnosed with MS, and one patient had a history of MS. Thirteen patients showed unremarkable results, and three patients showed other findings (one showed a finding of neurosarcoidosis, one showed global cortical atrophy, and one showed small punctuate focus of T2/FLAIR hyperintensity in both frontal lobes).

**Table 2:** Neurologic clinical manifestations.

Neurological symptoms	*n* = 45	%
Paresthesia	40	88.9
Numbness	39	86.7
Dizziness	39	86.7
Headache	36	80
Subjective cognitive decline	31	68.9
Subjective weakness	25	55.6
Imbalance	19	42.2
Neurological conditions diagnosed	n = 45	%
Peripheral neuropathy^a^	39	86.7
Small fiber neuropathy^b^	8	17.8
Dysautonomia^c^	10	22.2
Cognitive impairment^d^	12	26.7
Trigeminal neuralgia	3	6.7
Electroencephalogram abnormality^e^	2	4.4
Myelopathy^f^	1	2.2
Transient ischemic attack^g^	1	2.2

a Six patients had sensorimotor neuropathy and 33 had sensory neuropathy.

b Six patients showed positive skin biopsy. One patient was diagnosed based on clinical symptoms, evidence of small fiber neuropathy on a quantitative sensory test, and a normal nerve conduction test. One patient was diagnosed based on clinical symptoms, sudomotor dysfunction on autonomic tests, and a normal nerve conduction test.

c Ten patients were diagnosed using an autonomic test.

d Montreal Cognitive Assessment (MoCA) score ≤ 25/30. Of the 45 patients included in this study, 22 underwent MoCA testing.

e One patient had intermittent irregular delta activity in the left anterior-midtemporal region. One patient had intermittent focal theta to delta slowing in the left frontotemporal region.

f Cervical myelopathy with position sense loss.

g Likely due to a hypercoagulable state of unknown origin.

The reasons for neurology clinic referral are summarized in Table 3. The most common was neuropathy evaluation (*n* = 17, 37.8%), followed by consultations for abnormal MRI findings (n = 8, 17.8%), headache (*n* = 5, 11.1%), cognitive function (*n* = 5, 11.1%), and features of dysautonomia, particularly orthostatic symptoms (*n* = 4, 8.9%). One patient who was referred for myelopathy evaluation had position sense loss with electrophysiological evidence of spinal cord involvement. Of the eight patients who consulted for abnormal MRI findings, seven were referred to the neurology clinic with a concern of MS. Twenty-nine patients (64.4%) presented the neurology clinic prior to the diagnosis of seronegative pSS, with the median delay from the first neurology clinic presentation to the diagnosis being 5 (IQR 2.0–20.5) months.

**Table 3:** Reasons for neurology clinic referral.

Neurological condition	*n* = 45	%
Neuropathy	17	37.8
Magnetic resonance imaging abnormality	8	17.8
Headache	5	11.1
Cognitive dysfunction	5	11.1
Dysautonomia	4	8.9
Dystonia	1	2.2
Myelopathy	1	2.2
Seizure	1	2.2
Syncope	1	2.2
Transient ischemic attack	1	2.2
Multiple neurological conditions^a^	1	2.2

a Including bilateral hearing loss, right eye pain, weakness, dizziness, cognitive dysfunction, diffuse body pain, and headache.

The most common comorbidities observed were depression and migraine (*n* = 31, 68.9%). Twenty-one patients (46.7%) had been diagnosed as having fibromyalgia before presentation to our clinic, 13 patients (28.9%) had hypothyroidism, and 8 patients (17.8%) had irritable bowel syndrome.

Medication management is summarized in Table 4. Among the 45 examined patients, 36 had received at least one disease-modifying therapy. Thirty-one patients were on hydroxychloroquine, four were on steroids (one had a history of sarcoidosis, and one had a history of autoimmune hepatitis), two were on mycophenolate mofetil, and one was on methotrexate. Thirty-nine patients were on at least one medication for the treatment of neuropathic pain, including gabapentin (22 patients), duloxetine (20 patients), pregabalin (6 patients), venlafaxine (5 patients), baclofen (3 patients), and amitriptyline (1 patient). In addition, seven patients were on a selective serotonin reuptake inhibitor (three patients were on sertraline, two on fluoxetine, and one on escitalopram or citalopram). Thirty-seven patients had documented neuropathic symptoms at follow-up, of whom 25 (67.6%) had some symptomatic improvement after neuropathic medication initiation.

**Table 4:** Medication management of seronegative primary Sjögren’s syndrome with neurologic manifestations.

Treatment	*n* = 45
Immunotherapy (total)^a^	36
Hydroxychloroquine	31
Immunosuppressant	7
Steroid	4
Mycophenolate mofetil	2
Methotrexate	1
Treatment of neuropathic pain (total)^b^	39
Gabapentin	22
Duloxetine	20
Pregabalin	6
Venlafaxine	5
Baclofen	3
Amitriptyline	1

a Two patients were on more than one immunotherapeutic agent.

b Seventeen patients were on more than one medication for neuropathic pain.

## 4. Discussion

We described the demographic, neurologic manifestations, and treatment of patients with seronegative pSS who presented to the neurology clinic. Our findings regarding age and female-to-male ratio match previous reports (10); two patients had a family history of pSS. Although we could not find any genetic studies focusing on seronegative pSS, previous reports suggest a genetic predisposition to seropositive pSS with a complex mechanism involving both HLA and non-HLA genes (13). Most of these studies, however, discuss risk factors for the formation of anti-Ro/La antibodies, which may not apply to seronegative pSS. Further studies are needed to elucidate the link between seronegative pSS and genetic susceptibility.

In pSS, a variety of neurological problems have been reported (3–5), including peripheral neuropathy, which was the most common in our study, both from patients and as the documented reason for neurology referral (Tables 2, 3). While we did not compare seropositive and seronegative patients, other studies found that peripheral neuropathy was more prevalent in the seronegative group and had a greater impact on physical function outcomes (2, 9). Tani et al. (14) found that neuropathogenic effects mainly affected small nerve fibers instead of axon in seronegative pSS, which could explain the high frequency of paresthesia and small fiber neuropathy diagnosis in our patient cohort. The frequency of peripheral neuropathy in pSS patients ranges from 2 to 60%, depending on the detection methods used (5). The high proportion of neurologic conditions in our population is likely due to the study being conducted at a neurology clinic and including symptomatic patients regardless of quantitative assessments. Only 12 out of 31 patients reporting cognitive decline exhibited objective cognitive dysfunction. Previous research (15) showed that asymptomatic individuals with pSS displayed electrophysiological evidence of subtle cognitive dysfunction, while other studies indicated similar evidence of central nervous system dysfunction in asymptomatic patients with pSS using brainstem auditory evoked potentials and somatosensory evoked potentials (16, 17). This may explain part of the higher incidence of subjective neurologic clinical manifestations before a formal diagnosis is made.

MRI evaluation, particularly for concern of MS, was a common reason for neurology clinic referral. These concerns may be valid as pSS patients have a high prevalence of nonspecific T2WI/FLAIR white matter hyperintensities on brain MRI, even in the absence of focal neurologic symptoms (6, 18, 19). One study found that 49% of patients with pSS had white matter abnormalities, and 84% had multiple (≥3) lesions (19), which may lead to misdiagnosis of MS.

Several studies reported the relationship between migraine and pSS. Pal et al. (20) reported a significantly higher prevalence of migraine (46%) in patients with pSS. Escudero et al. (21) reported that the migraine-mimicking headache during pSS could be due to neurologic involvement and not just a comorbid migraine. Late-onset “migraine-like” episodes warrant evaluation to rule out pSS as a cause of headache. A previous study that demonstrated more severe physical function outcomes observed in seronegative pSS attributed the finding to concomitant fibromyalgia, which was more predominant in the seronegative pSS population (9). That study also reported that the fibromyalgia prevalence was twice as high in the seronegative group (33% vs. 17%). One large cohort study on the prevalence of fibromyalgia in pSS revealed a prevalence of 31% (22). Our study revealed that 46.7% of patients were documented to have fibromyalgia. This relatively high prevalence may reflect the symptom overlap between seronegative pSS and fibromyalgia, especially when patients have multiple symptomatic complaints, given that there is no specific laboratory examination to differentiate both conditions. Patients with seronegative pSS can be easily misdiagnosed or labeled with fibromyalgia and thus receive inappropriate treatment. Physicians should consider minor salivary gland biopsy in patients with suspected pSS, as serology can be negative in up to one-third of cases (1, 9).

Hydroxychloroquine is a commonly used immunomodulatory medication for musculoskeletal/joint pain associated with pSS (23). Although a randomized trial showed no significant improvement in pSS symptoms during 24 weeks of treatment (24), it is still commonly used as the first-line medication owing to its good safety profile and minimal side effects. All 40 patients with paresthesia received neuropathic pain medications, most commonly anticonvulsant calcium channel alpha 2-delta ligands, and serotonin-norepinephrine reuptake inhibitors for comorbid mood symptoms. Only seven patients received selective serotonin reuptake inhibitors for depression, highlighting the dual benefit of serotonin-norepinephrine reuptake inhibitors in managing chronic neuropathic pain, consistent with recommendations (25). Tricyclic antidepressants, due to anticholinergic side effects, were avoided and only used in one patient who had an allergic reaction to duloxetine. Symptomatic therapy for neuropathic pain led to decreased pain symptoms in 67% of patients, indicating the need for its consideration in seronegative pSS patients with neuropathic symptoms.

Our study has some limitations. It includes data from a single center with a high Caucasian predominance, reflective of the regional referral area’s racial and ethnic composition. Although the influence of race and ethnicity in this condition is still not well understood, one study from the Big Data Sjögren Project Consortium (an international multicenter registry that included 7,884 patients) also revealed a Caucasian predominance at 78.3% (10). Dedicated studies to determine if this accurately reflects racial/ethnic distribution or if other racial/ethnic groups are simply under-represented owing to healthcare disparities are beyond the scope of this retrospective review but are much needed in the future. Because this study was conducted in neurology clinics, our population had a high proportion of patients with neurologic complaints with a greater variety of neurologic symptoms than previously reported. While this study may not allow us to determine the frequency of neurologic complications in a general population, it aimed to characterize the clinical features in patients with seronegative pSS with neurologic involvement. Our study thus highlights the diversity of neurologic conditions seen in seronegative pSS and the importance of recognizing these manifestations, given their effect on patients’ quality of life. Owing to the nature of the retrospective chart review, incomplete medical chart documentation can affect the ability to comprehensively interpret the data; for example, one patient had a medical history of type 2 diabetes mellitus (no documented diabetic neuropathy), and four patients had a medical history of Hashimoto’s disease (data not presented). Similarly, the evaluation of treatment efficacy relied solely on the documentation provided by the treating physicians, with no utilization of standardized measurement tools, such as scales or scores. In comparison to existing literature, this study is among the largest to characterize the neurologic symptoms observed in patients with seronegative pSS and their response to treatment. Prospective clinical trials to evaluate neurologic symptoms and treatment outcomes in a multicenter setting would be more informative. Finally, the sensitivity of minor salivary gland biopsy as a diagnostic test varies between 60 and 86% (26, 27), and this represents an additional limitation. The specificity of minor salivary gland biopsy has, however, been reported to be relatively high (91–97%), making it useful for diagnosing pSS, especially in seronegative patients (26, 27). Our study included patients with Chisholm and Mason classification grades 3–4, which supports the diagnosis of seronegative pSS.

In summary, neurologic manifestations of seronegative pSS are heterogeneous and may precede or overshadow sicca symptoms, leading to difficulties in diagnosis, especially early in the course. Clinicians should be aware of the range of presentations of seronegative pSS and maintain a low threshold to perform diagnostic assessments, including minor salivary gland biopsy, especially since pSS without antibody positivity is common. It is also important to consider non-neurologic signs and symptoms—including sicca and joint pain—when evaluating patients with neuropathy to ensure that physicians do not miss a multisystemic disease such as pSS. This study also highlights that symptomatic therapy for neurologic symptoms in seronegative pSS is essential, as immunomodulatory therapy in isolation is rarely sufficient to manage symptoms and improve patient quality of life.

## Data availability statement

The raw data supporting the conclusions of this article will be made available by the authors, without undue reservation.

## Author contributions

YH: data acquisition, manuscript drafting, data analysis, and interpretation of the results. K-HW: data acquisition, conceptualization of the study, data analysis, interpretation of the results, and revising the manuscript for intellectual content. JGa, RB, JK, DL, JR, BT, CH, and JGr: revising the manuscript for intellectual content. SC: conceptualization of the study, data analysis, interpretation of the results, and revising the manuscript for intellectual content. All authors contributed to the article and approved the submitted version.

## Conflict of interest

The authors declare that the research was conducted in the absence of any commercial or financial relationships that could be construed as a potential conflict of interest.

## Publisher’s note

All claims expressed in this article are solely those of the authors and do not necessarily represent those of their affiliated organizations, or those of the publisher, the editors and the reviewers. Any product that may be evaluated in this article, or claim that may be made by its manufacturer, is not guaranteed or endorsed by the publisher.

## Abbreviations

FLAIR: fluid-attenuated inversion recovery
MRI: magnetic resonance imaging
MS: multiple sclerosis
pSS: primary Sjögren’s syndrome
RA: rheumatoid arthritis
T2WI: T2-weighted imaging.
